# Early and Complete Response of Bone Metastases, Documented by FDG-PET/CT Scan, in a Patient With NSCLC

**DOI:** 10.4021/wjon441w

**Published:** 2012-02-19

**Authors:** David Rossi, Paolo Giordani, Paolo Alessandroni, Vincenzo Catalano, Virginia Casadei, Anna Maria Baldelli, Stefano Luzi Fedeli, Francesco Graziano, Giammaria Fiorentini

**Affiliations:** a Azienda Ospedaliera “Ospedali Riuniti Marche Nord”, Presidio “San Salvatore” di Pesaro, Italy

**Keywords:** Gefitinib, NSCLC, PET/CT scan

## Abstract

In this case report we describe the case of a patient with multiple bone metastases of NSCLC, adenocarcinoma with exon 21 point-mutation of EGFR, treated with gefitinib. After only 3 months, FDG-PET/CT scan showed a complete response of bone metastases and right hylar adenopathy. Implications for need of early use of FDG-PET/CT scan after gefitinib treatment are discussed.

## Introduction

Bone metastases occured in 20 - 40% of patients with NSCLC and the prognosis is poor with a median overall survival of 7 months [1]. Chemotherapy and diphosphonates were the only therapeutic approach before the approval of gefitinib (IRESSA®; AstraZeneca) in patients with adenocarcinoma and EGFR-mutations, first in Japan/US and after in Europe. Two randomized phase III trials comparing gefitinib to chemotherapy as first-line treatment of patients with advanced NSCLC, selected by the presence of EGFR-mutation [2, 3]. Both trials demonstrated a statistically significant and clinically relevant increase in progression-free survival with gefitinib compared to platinum-based chemotherapy. Moreover, gefitinib seems to be a predictor of survival in patients with bone metaseases of lung cancer [4]. Other reports documented high activity of the drug in this setting of patients with long survival [5, 6]. Here, we describe the case of a female patient with mutilple bone metastases whose FDG-PET/TC scan became negative after only three months of treatment with gefitinib.

## Case Report

**Figure 1:**
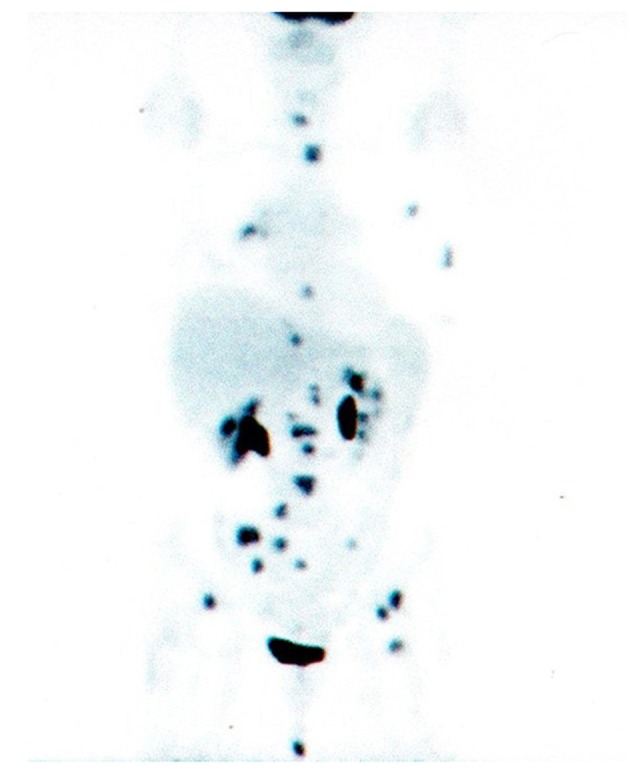
PET/CT scan before gefitinib treatment.

A 64-year-old, non-smoker, female patient was admitted for the first time to our Oncology Department on 1 July 2004. On 15 June 2004, she underwent right colectomy with diagnosis of adenocarcinoma, G3, pT3 N0 MO (Dukes B2, according Astler and Collins). We decided to start an adjuvant chemotherapy with iv folinic acid and 5-fluoruracile for 5 days (Machover schedule); on December 2004, the patient completed 6 cycles of treatment. Following clinical and instrumental controls were negative for disease relapse. On 9 March 2009, a Total-Body CT-scan, during follow-up, showed a nodule (38 × 35 × 29 mm) in the upper lobe of the right lung with lymphnodes on aorto-pulmonary window and Barety. Broncoscopy with aspiration was negative but FDG-PET/CT scan confirmed an uptake in the upper lobe of the right lung (SUVmax: 11.5) with other 3 uptakes: 1 on Barety (SUVmax: 4.4) and 2 on right lung hylum (SUVmax: 4.8). The patient decided to refer temporarly to another hospital. On May, the patient came back to our Institution: on 16 April 2009, she underwent right upper lobectomy and mediastinal adenectomy. The diagnosis was: “poor differentiated adenocarcinoma” (TTF1 +/-; Citocheratine 7 +++; CDX2 -) with metastasis on 11 lymphnodes (pT2 N2 M0 - stage IIIA). Considering the high risk of relapse, we decided to start and adjuvant chemotherapy with carboplatin/gemcitabine for 4 cycles (carboplatin AUC 4 on day 1 and gemcitabine 1000 mg/m^2^ on day 1 and 8, every three weeks). FDG-PET/CT scan performed on 8 October 2009 was negative. After 7 months, a Total-Body CT-scan showed a slight pleural effusion on right lung without other findings but two neoplastic markers were increased (CEA: 266 ng/ml; CA 19-9: 115 ng/ml). FDG-PET/CT scan performed on 17 June 2010, documented multiple uptakes on bones (ribs, dorsal and lumbar vertebrae, pelvis, left femur, left scapula, sacrum) and an uptake on right hylar adenopaty (Fig. 1). CEA: 311 ng/ml and CA 19-9: 148 ng/ml.

**Figure 2:**
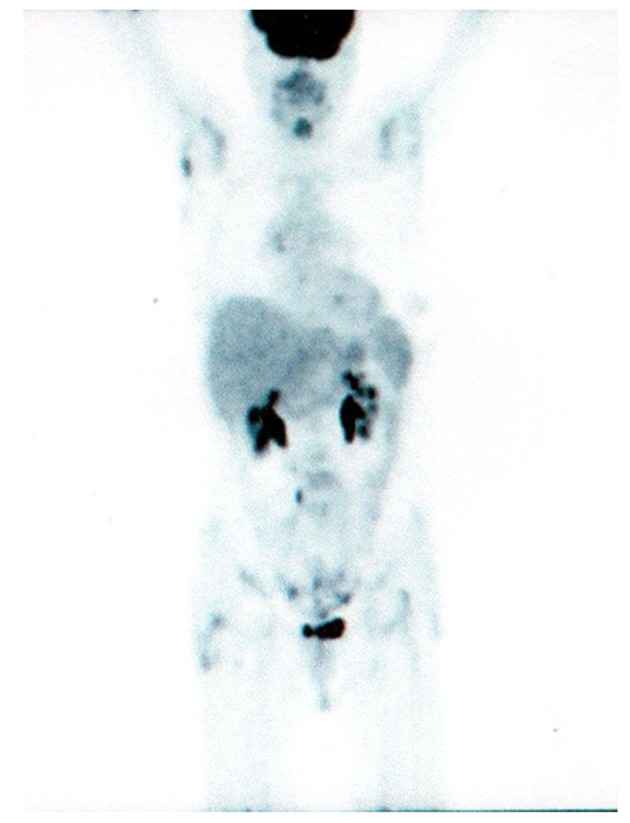
PET/CT scan after 3 months of gefitinib.

A search for EGFR-mutation was performed and iv zoledronic acid (4 mg every 4 weeks) was started. An exon 21 point mutation of EGFR has been found and the patient started gefitinib (IRESSA®; AstraZeneca) 250 mg/day on July 2010. FDG- PET/CT scan performed on 11 October 2010 showed the complete remission of all uptakes with reduction of neoplastic markers (Fig. 2). CEA: 21 ng/ml; CA 19-9: 13 ng/ml. The last clinical control was performed on 27 September 2011: the patient was in good condition without symptoms of note. During these 14 months of gefitinib treatment, the patient reported only 1 episode of grade 2 diarrhoea; a skin rash wasn't ever recorded. Total-body CT scan is negative and CEA value is 1.7 ng/ml.

## Discussion

Lung cancer has poor prognosis in patients with metastatic disease but only few reports have been published in patients with bone metastases treated with gefitinib; however, this drug seems to demonstrate high activity in this setting of patients [5-7]. Zukawa et al [5] reported two cases with remarkable bone formation of spinal metastases after gefitinib administration (without concomitant diphosphonates); Zampa et al [6] described the case of other two patients (1 with EGFR mutation and 1 without), whose bone-scan became negative after gefitinb treatment (with concomitant iv zoledronic acid) and long survival. Sugiura H et al [4] have tried to define some predictors of survival in patients with bone metastases of lung cancer and gefitinib was one of these. Despite the limitations due to small number of patients (52), the median survival was 17.8 months in the group treated with gefitinib (14 patients) and 10.8 months in the group withouot gefitinib (38 patients). These works seems to demonstrate that bone metastases of lung cancer are particularly sensitive to gefitinib treatment but, otherwise, there are mulecular explanations of these clinical findings. Normanno et al [8] demonstrated that gefitinib significantly inhibits the ability of bone marrow stromal to produce osteclastogenic factors, such as macrophage colony stimulating factors (M-CSF) and receptor activator of NF-kB ligand (RANKL). EGF-like ligands can regulate the expression of additional osteclastic regulatory factors in osteoblasts [9]. Certainly, other mechanisms might be involved in the control of disease progression on bones, such as neoangiogenesis: expression of the EGFR in the endothelial cells of experimental models of bone metastases has been documented [10]. Our case report describe the case of a female patient with multiple bone metastases of lung adenocarcinoma that harboring exon 21 point mutation of EGFR. After the disease progression, the patient started gefitinib at conventional dose (250 mg/day) with complete remission of all uptakes on bones (plus hylar adenopathy) documented by FDG-PET/CT scan. It's well established that FDG-PET/CT scan is more useful than Bone-Scan for evaluating metastases [11, 12]. Nevertheless, other biological data showed that, in gefitinib-sensitive cancer cells, EGFR kinase inhibition results in a rapid reduction of exogenous glucose utilization. This includes cells with EGFR kinase mutations (exon 21 point mutation or exon 19 deletion), as well as cells, with amplification of wild type EGFR. In contrast, gefitinib did not affect FDG uptake in resistant cell lines [13]. These findings suggest that FDG-PET/CT scan might be the best technique to predict tumor response after gefitinib administration, early in the course of therapy. We think that our report seems to confirm completely these findings, demonstrating the disapperance of all uptakes after only three months of treatment. Concomitant use of iv zoledronic acid might be crucial, cooperating to stop the tumor growth in the bone: a number of studies have demonstrated that zoledronic acid might blockade the growth of cancer cells [14]. Of course, other studies are warranted to investigate the real value of FDG-PET/CT scan in clinical practice to document early response of gefitinib treatment.
